# First Case of Robot-Assisted Surgery for Gynecologic Cancer at the Oncology Hospital, Centro Médico Nacional Siglo XXI

**DOI:** 10.7759/cureus.98911

**Published:** 2025-12-10

**Authors:** Rafael Medrano Guzman, Sabrina Carriles, Alvar J Vacio-Olguín, Moises Brener Chaoul

**Affiliations:** 1 Surgical Oncology, Oncology Hospital, Centro Médico Nacional Siglo XXI, Mexico City, MEX; 2 General Surgery, Hospital Angeles Lomas, Mexico City, MEX; 3 Oncological Surgery, Oncology Hospital, Centro Médico Nacional Siglo XXI, Mexico City, MEX; 4 Surgical Oncology, Hospital Angeles Lomas, Huixquilucan, MEX

**Keywords:** endometrial cancer, endometrioid adenocarcinoma, gynecological cancer, minimally invasive surgery, robotic-assisted surgery

## Abstract

Gynecologic malignancies represent a major public health challenge in Mexico, remaining among the principal causes of morbidity and mortality in women. Endometrial carcinoma constitutes one of the most frequent neoplasms, with its staging and management requiring a surgical approach in accordance with contemporary international standards. A descriptive report was conducted on the first patient treated with robot-assisted surgery for endometrial cancer at the Oncology Hospital. Preoperative assessment included standard imaging and clinical staging to confirm that the disease was confined to the uterus. The surgical procedure consisted of total hysterectomy with bilateral salpingo-oophorectomy and pelvic lymphadenectomy performed via robotic technique. Robot-assisted surgery represents a safe and feasible option for the management of early-stage endometrial carcinoma, offering advantages in terms of surgical precision, recovery time, and postoperative morbidity. This initial experience supports its incorporation as part of the comprehensive oncologic management within specialized centers.

## Introduction

The incidence of endometrial cancer has shown a significant increase; in Mexico, it ranks second among gynecologic neoplasms, with approximately 5,500 new cases and 1,100 deaths reported annually. This rise is largely attributable to the growing prevalence of obesity, which is the main risk factor. The risk increases in proportion to body mass index, which also contributes to various comorbidities [1,2]. These conditions limit the feasibility of the open surgical approach as a diagnostic-therapeutic protocol; minimally invasive is associated with reduced postoperative pain, shorter hospital stay, faster return to daily activities, and decreased intraoperative blood loss, making it an advantageous option [3,4]. Robotic surgery represents a technological advance that has consolidated the benefits of minimal invasion in the treatment of gynecologic cancer, mainly in pathologies such as endometrial and cervical cancer [5-7]. Robotic platforms provide three-dimensional magnified visualization, tremor filtration, and articulated instruments with seven degrees of freedom, allowing for enhanced precision and dexterity. These features facilitate complex dissections, especially in patients with obesity, severe endometriosis, or extensive pelvic adhesions [3,4]. Several international studies have reported its superiority in surgical parameters compared to laparotomy and conventional laparoscopy [8,9].

The robotic platform offers advantages such as enhanced three-dimensional vision, greater precision, elimination of physiological tremor, movement scaling, surgical ergonomics, and wrist-like instruments with seven degrees of freedom that mimic human wrist mobility [10]. These features allow for meticulous dissection, improving the safety and effectiveness of the procedure [6,11]. Additionally, current evidence suggests that the use of this technology does not compromise oncologic outcomes, such as disease-free survival and overall survival, which also depend on histopathological factors such as tumor size, myometrial invasion, lymphovascular involvement, and nodal metastasis [2,12].

Over the last two decades, minimally invasive surgery, including robot-assisted laparoscopy, has been progressively implemented in gynecologic oncology, being associated with reduced blood loss, fewer postoperative complications, and faster recovery compared to laparotomy [9,11]. The UMAE (High Specialty Medical Unit) Oncology Hospital, Centro Médico Nacional Siglo XXI, Instituto Mexicano del Seguro Social, is a specialized unit dedicated exclusively to the care of cancer patients and is the only one in the institute that currently has a robotic surgery platform. Reporting this case will help demonstrate its feasibility in a developing healthcare setting [3].

## Case presentation

This report describes one of the earliest gynecologic cancer cases managed with robot-assisted surgery at our institution. The patient was a 49-year-old woman, originally from and residing in Mexico City, with a two-year history of systemic arterial hypertension, no prior surgeries, obesity (BMI 31), and no personal or family history of cancer. She denied smoking, alcohol consumption, and allergies.

Regarding her gynecologic and obstetric history, she reported menarche at age 12, regular cycles, onset of sexual activity at age 18 with one sexual partner, Gravida 4, Para 4, and bilateral tubal ligation as her contraceptive method. She denied the use of hormonal contraceptives.

**Figure 1:**
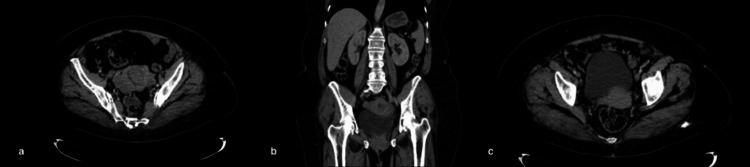
Preoperative computed tomography. (a) Axial view showing a solid-appearing lesion occupying the endometrial cavity, with increased density and distortion of the uterine architecture. (b) Coronal view demonstrating a tumor confined to the uterine body, with no evidence of invasion into adjacent pelvic structures. (c) Inferior axial view depicting a clear delineation of the uterus and absence of adnexal masses or pelvic lymphadenopathy.

The current condition began two years before she consulted with progressive postmenopausal vaginal bleeding. A transvaginal ultrasound showed a thickened endometrium (10.2 mm) with normal-appearing ovaries and cervix. Uterine curettage with endometrial biopsy revealed well-differentiated invasive endometrioid adenocarcinoma. Staging studies with chest, abdominal, and pelvic CT scans, performed as the institutional standard, showed no evidence of extrauterine disease (Figure 1).

**Figure 2:**
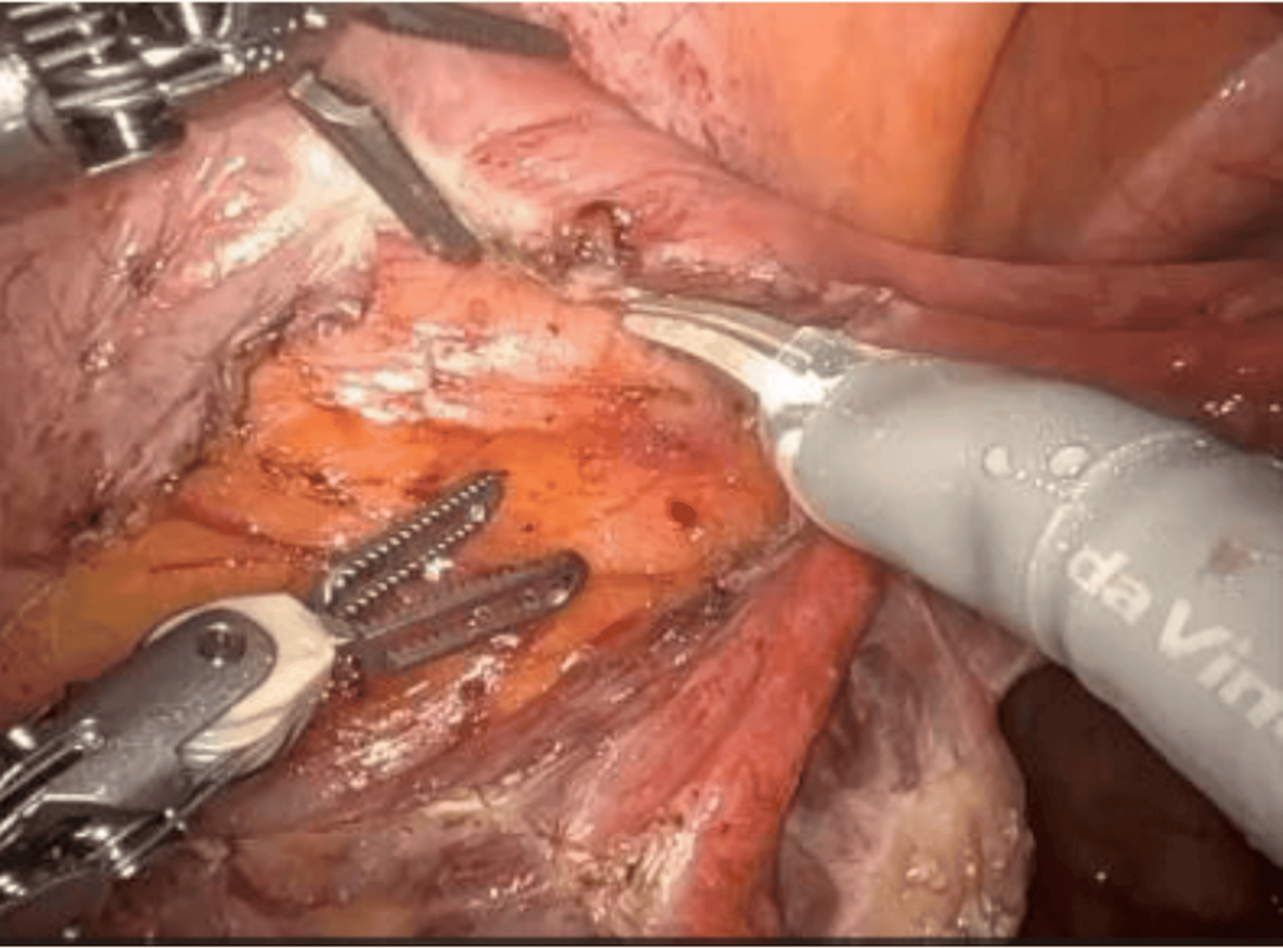
Robotic approach to pelvic lymphadenectomy. Intraoperative images were published with the patient's consent. Opening of the lateral peritoneum to expose the infundibulopelvic ligament.

**Figure 3:**
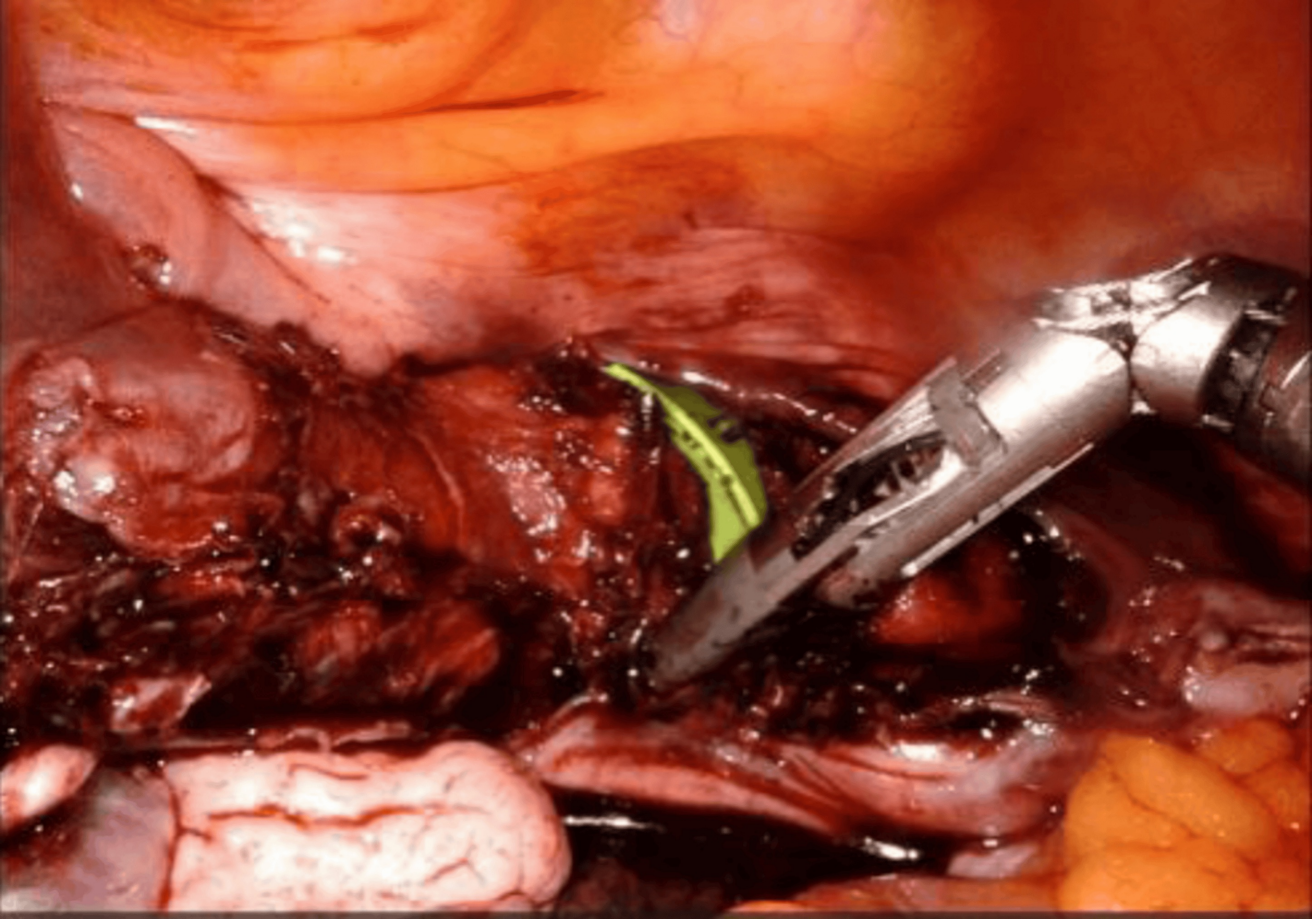
Vaginal incision by robotic surgery. Intraoperative images published with the patient’s consent, showing the opening and circumferential sectioning of the vagina around the uterine manipulator (shown in green).

**Figure 4:**
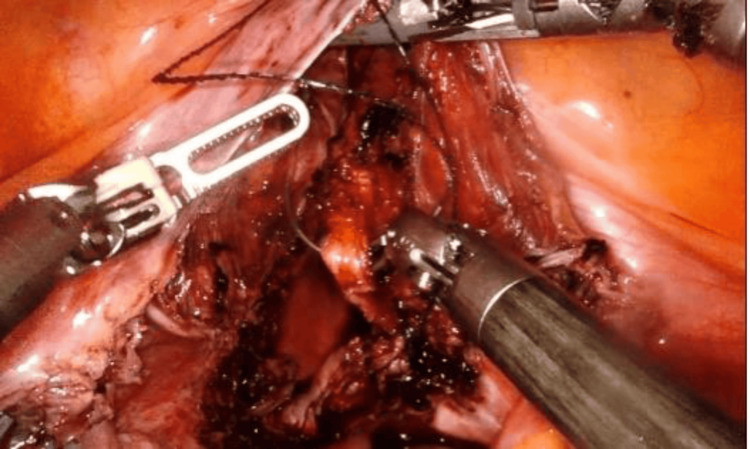
Vaginal vault closure by robotic surgery. Intraoperative images published with the patient’s consent, showing the vaginal cuff being approximated and closed using V-LOC 2-0 sutures.

On December 22, 2022, a staging surgery was performed via robot-assisted laparoscopy, including a Piver type I total hysterectomy, bilateral salpingo-oophorectomy, and bilateral pelvic lymphadenectomy. Using the Da Vinci Xi system, a supraumbilical port and three 8 mm trocars were used (one on the left flank, two on the right flank). The procedure was uneventful, with an estimated blood loss of 10 cc, an operative time of 180 minutes, and hospital discharge at 24 hours (Figures 2-4).

Histopathological analysis revealed moderately differentiated endometrioid adenocarcinoma, measuring 5 x 4.5 cm, FIGO grade 2, confined to the endometrial cavity, with less than 50% myometrial invasion. There was no endocervical or parametrial invasion; lymphovascular invasion was present, with no perineural invasion. A total of 10 pelvic lymph nodes were resected, all negative for malignancy. Other findings included chronic cystic cervicitis.

Based on these findings, the disease was classified as early stage, with no indication for adjuvant therapy. To date, the patient has remained under follow-up with regular physical examinations and CT scans, with a disease-free period of 30 months and no evidence of local or distant recurrence.

## Discussion

Minimally invasive robot-assisted surgery for early-stage endometrial cancer has been shown to provide oncological outcomes equivalent to those of open surgery, while offering additional benefits. Eoh et al. reported higher estimated blood loss in the laparotomy group compared with the robotic surgery group (316.8 cc vs. 113.0 cc; *P* < 0.010). Robot-assisted surgery was also associated with less postoperative pain. Additionally, He et al. found that robotic surgery resulted in a shorter length of hospital stay (mean difference (MD), -3.42; 95% confidence interval (CI), -3.81 to -3.03; *P* < 0.01) and lower rates of postoperative complications (odds ratio (OR), 0.62; 95% CI, 0.52-0.73; *P* < 0.01) compared with laparotomy [5,9,11]. In this first case treated at our institution, the feasibility and safety of the technique were confirmed, with an operative time of 180 minutes, a hospital stay of only 24 hours, and minimal blood loss, consistent with findings reported in international series [6,8]. The use of the robotic platform enabled precise and ergonomic dissection, facilitating anatomical access and preserving critical structures, aspects that are particularly relevant in patients with comorbidities such as systemic arterial hypertension.

Furthermore, the oncological control achieved at 30-month follow-up, assessed by physical examinations during clinical visits and CT scans, supported the notion that incorporating this technology did not compromise disease-free survival, as reported in previous studies [2,7,12]. The absence of adjuvant treatment requirements in this case reflects the importance of timely diagnosis and accurate surgical staging. According to the clinical practice guidelines of the Mexican Social Security Institute, low-risk patients do not require adjuvant treatment; that is, patients under 60 years of age with any type of invasion or patients of any age with grade 3 invasion [13]. Although the present experience is limited to a single case, these results establish a precedent for the progressive implementation of robotic surgery in the management of gynecologic cancer within the Mexican Social Security Institute, opening the door to prospective studies that may evaluate its cost-effectiveness, accessibility, and applicability in diverse clinical settings. In Mexico, of 58 public tertiary-level hospitals, only 10 have robotic surgery systems, and only one of these systems belongs to the Mexican Social Security Institute [14].

## Conclusions

This first case demonstrates the benefits of incorporating robot-assisted surgery in the treatment of gynecologic cancer at the Oncology Hospital, Centro Médico Nacional Siglo XXI. The experience gained from this initial case provides a strong foundation for expanding the use of robotic surgery in selected gynecologic oncology patients within the institution, potentially leading to standardized protocols that enhance surgical precision, reduce morbidity, and optimize patient-centered outcomes. As robotic platforms become more accessible and surgical teams gain proficiency, this technology has the potential to redefine the standard of care for gynecologic cancers in Mexico.
